# Kentucky Surgery

**Published:** 1850-02

**Authors:** 


### KENTUCKY SURGERY.

It is pretty generally known in this country, that whatever credit is due for the introduction of the operation of exsection of the human ovary, belongs to a Kentucky surgeon—Dr. Ephraim M'Dowell. Mr. Lizans attempted to rob the Kentuckian of this honor, but the evidence adduced by Dr. M'Dowell in favor of his priority, was conclusive.

In the New Orleans Journal of Medicine and Surgery, we find a paper from the pen of James H. Johnson, M. D., of New Orleans, late Professor of Surgery in Franklin Medical College of Missouri, which conclusively establishes the fact, that a Kentucky physician preceded Dr. Mott in "the exsection of the clavicle, for osteo-sarcoma," a piece of surgery, which Dr. Mott has dignified with the title of "Mott's Waterloo Operation," and says he claims the credit of it "for his country, his city, and himself." But Dr. Johnson establishes the fact that Dr. Charles M'Creary, of Hartford, Kentucky, preceded Dr. Mott, in this operation, fully seventeen years. Dr. Mott reported his case in the "American Journal of the Medical Sciences," for November, 1828, and Dr. M'Creary had exsected the clavicle, for osteo-sarcoma, on the 4th day of May, 1811, and Dr. Johnson says: "The method adopted by Dr. M'Creary was almost similar to Prof. Mott's."

If Dr. Mott thought proper to claim credit for "his city and himself" for originating the operation referred to, it cannot be improper for us to vindicate the claim of a Kentucky surgeon to this originality. The Kentuckian deserves more credit than Dr. Mott could reasonably claim, for the New Yorker, in 1828, had all the appliances for such an operation, as the exsection of the clavicle, in a degree of much greater perfection, than the Kentuckian had in 1811.

